# Enzyme Release from Polyion Complex by Extremely Low Frequency Magnetic Field

**DOI:** 10.1038/s41598-020-61364-w

**Published:** 2020-03-16

**Authors:** Kseniya Yu. Vlasova, Hemant Vishwasrao, Maxim A. Abakumov, Dmitry Yu. Golovin, Sergey L. Gribanovsky, Alexander O. Zhigachev, Andrey А. Poloznikov, Alexander G. Majouga, Yuri I. Golovin, Marina Sokolsky-Papkov, Natalia L. Klyachko, Alexander V. Kabanov

**Affiliations:** 1 Laboratory for Chemical Design of Bionanomaterials, School of Chemistry, Lomonosov Moscow State University, Moscow, 119991 Russia; 2 Center for Nanotechnology in Drug Delivery and Division of Molecular Pharmaceutics, Eshelman School of Pharmacy, University of North Carolina at Chapel Hill, Chapel Hill, NC 27599 USA; 3 National University of Science and Technology MISIS, Moscow, 119049 Russia; 4 Department of Medical Nanobiotechnology, Pirogov Russian National Research Medical University, Moscow, 117997 Russia; 5 G.R. Derzhavin Tambov State University, Tambov, 392036 Russia; 6 FSBI National Medical Research Radiological Center of the Ministry of Health of the Russian Federation, Obninsk, 249036 Russia; 7 D. Mendeleev University of Chemical Technology of Russia, Moscow, 125047 Russia

**Keywords:** Biotechnology, Chemistry, Biomaterials, Nanoscale materials, Nanoscience and technology, Nanobiotechnology, Nanomedicine

## Abstract

Remote nano-magneto-mechanical actuation of magnetic nanoparticles (MNPs) by non-heating extremely low frequency magnetic field (ELF MF) is explored as a tool for non-invasive modification of bionanomaterials in pharmaceutical and medical applications. Here we study the effects of ELF MF (30–160 Hz, 8–120 kA/m) on the activity and release of a model enzyme, superoxide dismutase 1 (SOD1) immobilized by polyion coupling on dispersed MNPs aggregates coated with poly(L-lysine)-*block*-poly(ethylene glycol) block copolymer (s-MNPs). Such fields do not cause any considerable heating of MNPs but promote their rotating-oscillating mechanical motion that produces mechanical forces and deformations in adjacent materials. We observed the changes in the catalytic activity of immobilized SOD1 as well as its release from the s-MNPs/SOD1 polyion complex upon application of the ELF MF for 5 to 15 min. At longer exposures (25 min) the s-MNPs/SOD1 dispersion destabilizes. The bell-shaped effect of the field frequency with maximum at *f* = 50 Hz and saturation effect of field strength (between 30 kA/m and 120 kA/m at *f* = 50 Hz) are reported and explained. The findings are significant as one early indication of the nano-magneto-mechanical disruption by ELF MF of cooperative polyion complexes that are widely used for design of current functional healthcare bionanomaterials.

## Introduction

Non-invasive functional control of bionanomaterials in inaccessible areas of a human body (e.g., biochemical reactions, drug release, gene expression, etc.) could greatly advance development of diagnostics and therapeutic modalities^1^. This control can be accomplished by remote actuation of nanoparticles by external fields^2–6^. In recent years magnetic nanoparticles (MNPs) have been investigated for cell separation, targeted drug delivery, and magnetic resonance imaging (MRI)^7–9^. Remote actuation of MNPs by radio frequency (200–800 kHz) magnetic fields has been used in hyperthermia for cancer therapy^10–12^. However, this approach has a risk of tissue overheating and damage. Recently an alternative nano-magneto-mechanical actuation of MNPs by non-heating extremely low frequency magnetic field (ELF MF) has attracted increasing attention in drug delivery and nanomedicine^13–17^.

The phenomenon of nano-magneto-mechanical actuation is linked to the ability of MNPs to undergo mechanical motion in ELF MF and as a consequence produce mechanical forces and deformations in adjacent materials. It is well known, that a single-domain MNPs can relax in an external magnetic field, reducing an angle between the MNPs magnetic moment ***μ*** and the field vector ***H***^18–20^. Two basic types of relaxation phenomena are dependent on the size of the magnetic core. MNPs with *D*_*m*_ less than some critical value d* experience rotation of magnetic moments ***μ*** towards the vector ***H***, which proceeds mainly by overcoming the crystallographic anisotropy of the lattice, without causing significant mechanical motion of the particle (Néel relaxation). MNPs with a diameter *D*_*m*_ greater than d* dissipate their energy in alternating magnetic field mainly by mechanical rotation (Brown relaxation). For commonly used magnetite MNPs d* ~ 13 nm^19,20^ and their transition to a multi-domain state occurs at sizes of about 120 nm^7^. Detailed theoretical examination of the principles of non-thermal nano-magneto-mechanical actuation and the optimization of MNPs and magnetic fields for biochemical applications were previously described^16,17^. The experimental validations of this phenomenon reported so far appear to provide support for the ability of MNPs to disrupt well-defined molecular structures, such as protein globule conformation, “lock and key” protein complex, lipid bilayer packaging or cell cytoskeleton networks. For example, magnetic field-responsive biocatalytic systems with enzyme molecules or enzyme-inhibitor complex attached to MNPs were shown to change catalytic activity in ELF MF^21,22^. Several studies have focused on applying ELF MF to increase the permeability of magnetic liposomes for dye or drug release^23^. Other studies reported nano-magneto-mechanical induction of stem cells differentiation as well as cytoskeletal destruction of and cytotoxicity in cancer cells^24,25^. At the same time very widely used principle of design of current bionanomaterials involves much less defined although highly cooperative and very strong polyion complexation of oppositely charged polyelectrolytes^26^. This paper for the first time describes the release of the enzyme, superoxide dismutase 1 (SOD1), from the polyion complex formed by electrostatic complexation of this enzyme with a cationic polymer coated MNPs via activation by non-heating ELF MF.

## Results

The MNPs were synthesized by thermal decomposition of Fe(acac)_3_ in benzyl alcohol. The resulting nanoparticles were nearly spherical with the mean diameter *D*_*m*_ of ~9 nm (Supporting information, Figure S1) and contained at least 80% of Fe_3_O_4_ vs. no more than 20% γ-Fe_2_O_3_ according to the Mössbauer spectroscopy (Supporting information, Figure S2). The MNPs were then coated with the cationic block copolymer, poly(L-lysine)-*block*-poly(ethylene glycol) (PLL-PEG) in aqueous media to produce stable dispersions as described in Experimental section. As seen in Table 1 the block-copolymer stabilized MNPs (s-MNPs) represent aggregates of from ~80 to ~100 nm in effective hydrodynamic diameter (*D*_*HD*_), polydispersity indexes (PDI) ranging from 0.23 to 0.29 and high positive ζ-potential ranging from ∼27 to ∼45 mV. They show little dependence of these characteristics on the length of the polycation block of PLL-PEG. The magnetite content in the s-MNPs as measured by the thermogravimetric analysis (TGA) was in the range of 45 to 55% by weight, with the rest being the block copolymer. The coating by the block copolymer did not affect significantly the magnetic properties of the MNPs. Specifically, the saturation magnetization normalized for the magnetite mass was 58 emu/g and 57 emu/g for bare MNPs and s-MNPs respectively (Supporting information, Figure S1), which is consistent with the literature data for spherical magnetite MNPs with *D*_*m*_ = 10 nm^27^.

**Table 1:** Physicochemical characteristics of stabilized s-MNPs and their complexes with SOD1^a^.

Sample	*D*_*HD*_, nm	PDI	ζ-potential, mV
MNP/PLL_10_-PEG	84 ± 12	0.24 ± 0.07	36.5 ± 0.3
MNP/PLL_50_-PEG	82 ± 12	0.23 ± 0.01	38.0 ± 1.0
MNP/PLL_100_-PEG	98 ± 10	0.29 ± 0.04	44.5 ± 2.0
MNP/PLL_50_-PEG/SOD1	108 ± 2	0.20 ± 0.03	29.8 ± 0.5
MNP/PLL_100_-PEG/SOD1	113 ± 3	0.19 ± 0.01	27.1 ± 1.0

^a^All measurements were carried out at RT in 0.1 mM HEPES buffer, pH 7.4. For all s-MNPs samples the Fe_3_O_4_ concentration of was 10 µg/mL. For s-MNPs/SOD1 complexes the Fe_3_O_4_ concentration was 11.5 µg/mL. Data are presented as mean ± SD (n = 3).

The s-MNPs aggregates bear positive charge due to the presence of an excess of the cationic PLL chains of PLL-PEG in these aggregates. Since SOD1 molecule at pH 7.4 is negatively charged (ξ-potential - 7 mV, isoelectric point pI 5.35–6.75, *D* = ∼5.0 nm), it was immobilized on s-MNPs by simple polyion complexation. The s-MNPs obtained using PLL_10_-PEG rapidly aggregated and precipitated upon addition of the enzyme (Supporting information, Figure S3), and they were not studied further. In contrast, the s-MNPs obtained using two other copolymers with longer PLL blocks (50 and 100 repeating units (r.u.)) formed stable s-MNPs/SOD1 complexes. These complexes were separated from non-incorporated SOD1 by triple centrifugal filtration until no free enzyme was detected in the solution by the Bradford method. The purified s-MNPs/SOD1 complexes (Table 1) were also stable in the solution and did not aggregate for over 7–10 days. The particles in these dispersions still exhibited positive charge albeit their ζ-potentials considerably decreased after addition of SOD1 compared to the initial s-MNPs (Table 1). Interestingly, the size, polydispersity, and ζ-potentials of the s-MNPs complexes with SOD1 exhibited little dependence on the molar ratio of the PLL amino groups to SOD1 carboxyl groups (Table 2). It appears that independent of this ratio, the amount of the enzyme immobilized on the s-MNPs was approximately the same, which may reflect a saturation of SOD1 binding sites in the s-MNPs at all ratios used.

**Table 2:** Physicochemical characteristics of the purified MNPs/PLL_100_-PEG/SOD1 complexes at various charge ratios^a^.

Molar ratio of PLL amino groups to SOD1 carboxyl groups during the complex formation	2:1	1:1	1:2
*D*_*HD*_, nm	110 ± 12	116 ± 6	104 ± 8
PDI	0.22	0.37	0.26
ξ-potential, mV	27 ± 5	30 ± 7	25 ± 8
SOD1/MNPs ratio, µg SOD1/µg Fe_3_O_4_	0.281 ± 0.014	0.294 ± 0.015	0.228 ± 0.014

^a^The DLS measurements were carried out at RT in 0.1 mM HEPES buffer, pH 7.4. For all samples the Fe_3_O_4_ concentration of was 10 µg/mL. The SOD1 and Fe_3_O_4_ contents were determined by ICP-MS.

**Figure 1:**
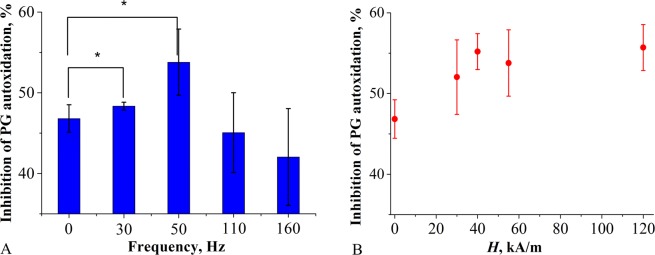
Effect of ELF MF on the SOD1 enzymatic activity of the MNPs/PLL_100_-PEG/SOD1 complex at **(A)** the constant field intensity of 55 kA/m and varying field frequency and **(B)** the constant field frequency of 50 Hz and varying field intensity. The samples were exposed to the ELF MF for 30 sec at RT. The Fe_3_O_4_ and SOD1 concentrations were 50 ng/mL (or 54.6 ng/mL **(B)**) and 48 ng/mL (or 50 ng/mL **(B)**), respectively. The samples were dispersed in 50 mM Tris-HCl buffer, pH 8.2. Data are presented as mean ± SD (n = 3); *p ≤ 0.05.

Immobilization of SOD1 on s-MNPs resulted in approximately two-fold decrease in the catalytic activity compared to the native SOD1 (Supporting information, Figure S4). Next, we examined the effect of ELF MF exposure on the activity of s-MNPs/SOD1 complexes. To exclude any interference of ELF MF with the radical chemical reaction of pyrogallol (PG) autoxidation the substrate was added to the complexes after their exposure to the field and the rates of PG autoxidation were measured immediately thereafter^28^. Also, we confirmed that the ELF MF had no influence on the activity of the free SOD1 or PG autoxidation (Supporting information, Figure S5 and S6). The MNPs/PLL_50_-PEG/SOD1 complex was not responsive to the effect of the ELF MF (Supporting information, Figure S7). In contrast, exposure of the MNPs*/*PLL_100_-PEG/SOD1 complex to ELF MF resulted in the enzyme activity increases. Interestingly at the constant field strength (55 kA/m) the magnitude of the effect depended on the field frequency and was the most pronounced at *f* = 50 Hz (Fig. 1A). Likewise, at constant field frequencies the effect depended on the field strength and appeared to “saturate” between 30 kA/m and 120 kA/m at *f* = 50 Hz (Fig. 1B; Supporting information, Figure S8).

**Figure 2:**
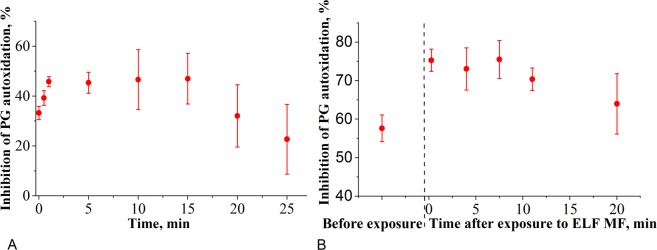
Dependence of the MNP-PLL_100_-PEG/SOD1 activity on the **(A)** duration of exposure of this complex to the ELF MF (*f* = 50 Hz, *H* = 55 kA/m) or **(B)** time elapsed after 5 min. exposure of this complex to the field. The samples were dispersed in 50 mM Tris-HCl buffer, pH 8.2. **(A)** Filled circles and squares correspond to two different samples preparations. **(A, B)** Data are presented as mean ± SD (n = 3). **(А)** The Fe_3_O_4_ and SOD1 concentrations were 43 ng/mL and 28.5 ng/mL, respectively. **(B)** The Fe_3_O_4_ and SOD1 concentrations were 54.6 ng/mL and 50 ng/mL, respectively.

The enzyme activity also depended on the duration of the exposure of MNPs/PLL_100_-PEG/SOD1 to the field (Fig. 2A). During approx. 5 min of the exposure there was a trend for the growth of the SOD1 activity, then it was levelled off and then, after 15 min, displayed a tendency to decrease. When the complex was exposed to the field for 5 min and left without the field the activity decreased nearly back to the level observed before the field exposure (Fig. 2B), i.e. the effect of the field was reversible. Therefore, we observed a complex behaviour of the MNPs/PLL_100_-PEG/SOD1 activity during and after ELF MF exposure.

We posited that upon short-term exposure to ELF MF SOD1 desorbed from the complex, which was accompanied by the activity increase. To test this we separated the complexes by centrifugal filtration before and at different time points after the field exposure using cellulose filters with pore sizes permeable to proteins with a mass of less than 100 kDa but impermeable for the complexes proper. After 5 min exposure to ELF MF (*f* = 50 Hz, *H* = 55 kA/m) there was ∼5-fold increase in the SOD1 protein in the filtrate (in equal volumes of the solutions) as determined by electron ionization mass spectroscopy (EI MS) (Table 3) and nearly two-fold increase in the rate of the PG autoxidation catalyzed by SOD1 (Supporting information, Table S1). Interestingly, along with the increase in the amount of the enzyme in the filtrate there was also an increase in the amount of the block copolymer as shown by EI MS (Table 3). Therefore, during a short-term exposure both SOD1 and PEG-PLL are partially stripped from the complex. At longer exposures such as 25 min the amount of SOD1 detected in the filtrate decreased. This may be due to reabsorption of the enzyme at the vessel or destabilization of the whole system due to polycation desorption and aggregation leading to conditions at which the enzyme becomes bound to the aggregates.

**Table 3:** Release of SOD1 and PLL-PEG in the filtrated after the exposure of the MNP-PLL_100_-PEG-SOD1 complex dispersion to ELF MF (*f* = 50 Hz, *H* = 55 kA/m).

Analyte in the filtrate	Relative peak intensities, normalized to the control		
	No ELF MF	5 min ELF MF^a^	25 min ELF MF^a^
SOD1	1.0	5.6 ± 2.5	1.5 ± 0.8
PLL-PEG	1.0	2.4 ± 1.0	0.9 ± 0.3

^a^During the exposure of the MNP-PLL_100_-PEG-SOD1 dispersion to the ELF MF the Fe_3_O_4_ and SOD1 concentrations were 43.6 μg/mL and 49.2 μg/mL, respectively. Following the exposure to the magnetic field the MNP-PLL_100_-PEG-SOD1 samples were concentrated via centrifugal filtration (cut-off 100 kDa). The filtrates were analyzed by EI MS for SOD1 and PLL_100_-PEG (Supporting information, Figure S10). The values of the peak intensities were normalized to the control that was not exposed to the ELF MF. Data are presented as mean ± SD (n = 3). The SOD1 concentrations in the in filtered MNP-PLL_100_-PEG-SOD1 samples as determined by ICP-MS were 22.4 ± 0.4 μg/mL, 18.8 ± 0.1 μg/mL and 23.2 ± 0.1 μg/mL before, and after 5 min and 25 min exposure to the magnetic field.

During a short-term (5 to 15 min) exposure of the complex to the field there were no changes in the particle size or polydispersity (Supporting information, Figure S9). In contrast, at the longer exposure (25 min) a considerable aggregation and increase in PDI were observed. Evidently in the absence of the field the s-MNPs systems are stabilized in the dispersion by steric repulsion of the block copolymer chains bound to the MNPs, which effectively increase the distance between the magnetic cores in the separate nanocomplexes. However, in the presence of ELF MF due to desorption of the block copolymer chains the nanocomplexes may become less stable. Moreover, continuous exposure to the magnetic field should enhance a tendency of MNPs to line up in linear aggregates^29,30^.

## Discussion

We have demonstrated that during ELF MF exposure the polyion complexes of a cationic polymer coated MNPs with electrostatically immobilized SOD1 can undergo reversible desorption of the enzyme from the complex. The released enzyme has greater catalytic activity than the enzyme immobilized in the complex. We have shown that the SOD1 activity in the system depends on the duration of the exposure of the complexes to the ELF MF, as well as on the field strength and frequency.

A detailed consideration of a possible mechanism of macromolecules desorption from the MNPs oscillating under ELF MF were given in refs. ^16,31^. The phenomenon is of non-thermal nature since the local or system-wide heating produced by ELF MF (***f*** < 1 kHz, ***H*** < 100 kA/m) during the observation times is negligible (see Supporting Information). Briefly, an external homogeneous alternating magnetic field produces rotational motion of MNPs with the amplitude of the swept angle ***Δφ***, depending on the MNP hydrodynamic size *D*_*HD*_, the intensity ***H*** and the frequency ***f*** of the magnetic field, as well as the viscosity of the environment ***η***. The value of ***Δφ*** tends to 180 ° in the limit for small *D*_*HD*_, ***η*** and ***f***. The optimal range of sizes of magnetite MNPs *D*_*m*_ for the most effective conversion of magnetic field energy into mechanical motion is ~ 30–200 nm. The optimal frequency of the MF (at which the value of ***Δφ*** is close to 180°) ranges from fraction of Hz to kHz (depending on *D*_*HD*_ and ***η***). Since s-MNPs consist of clusters of multiple particles, the entire clusters will be mechanically moving, and not the individual particles. As follows from the dynamic light scattering (DLS) data, binding of SOD1 to s-MNPs clusters practically does not change the size the clusters. Thus, we can assume that the case of the enzyme, “sandwiched” between two or more cluster particles is avoided.

**Scheme 1:**
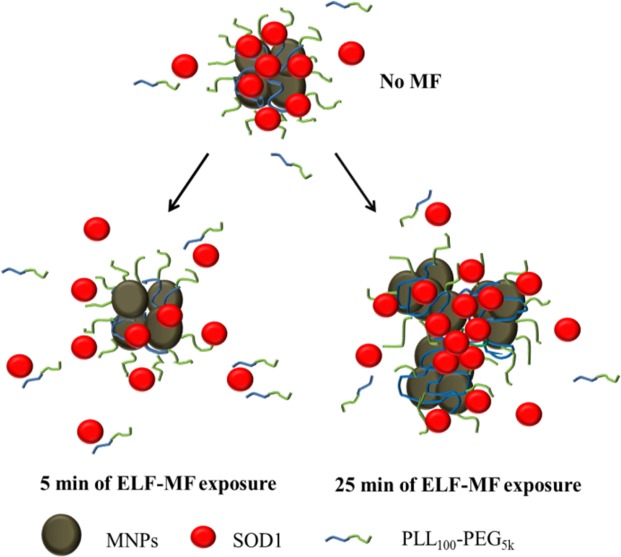
Schematic representation of the effects of ELF MF on the s-MNPs/SOD1 complex.

For a SOD1 protein globule with *D*_*g*_ ~ 5 nm, bound to s-MNP surface, in the media with ***η*** ≈ 10^−3^ Pa∙s the amplitude peak value of hydrodynamic force acting upon this globule, *F*_*HD*_ would be in the order of tenth of pN^31^. This is a very small force, which in itself cannot overcome cooperative electrostatic and Van der Waals interactions between species in the polyion complex. However, this force can produce a slope in the potential profile of SOD1 interaction with the polycation chains directed from the s-MNPs cores to the periphery. Since there is excess of the amino groups of PLL vs. carboxylic groups of SOD1 in s-MNPs/SOD1 complexes the SOD1 molecule can interact with neighbouring free cationic groups and polycation segments and thereby change its relative position in the corona of the s-MNPs. This process is similar to the well-known polyion interchange reactions in the polyion complexes involving displacement of one polyion chain for another^32^. These reactions proceed as a result of fluctuations when the reacting polyions interpenetrate each other due to the thermal motion. Under the effect of thermal fluctuations SOD1 can leave its potential energy level and move to the next one. It is known that the migration of a polyion in a weakly cross-linked network of opposite charge can display spatial directionality, controlled by a gradient of pH, ionic strength, etc^32^. Although the mechanistic basis underlying the effects of ELF-MF in this context cannot fully be accounted for on a theoretical basis, we posit that in our case due to the rotational-vibrational motion of s-MNPs under ELF MF, the bottom of the energy profile inclines by the action of hydrodynamic force *F*_*HD*_ on the reaction. It is crucial that this inclination is always directed from the s-MNP, although it changes its value over time during ELF MF oscillation. At sufficiently high polycation chain length (and hence the number of local minima *n* in the potential profile), even a slight difference in probability of hopping inward and outward can significantly accelerate the diffusion of the SOD1 from the core to the periphery of the s-MNPs corona. Once the SOD1 molecule reaches periphery in the s-MNPs corona the hydrodynamic force cannot ensure its further release in the external solution. However, since we detect the excess of the polycation in the solution the enzyme can be removed from the s-MNPs/SOD1 complex in the external solution by displacement into a complex with the free polycation by polyion interchange mechanism (Scheme 1). Although we use term “desorption” to describe the observed phenomenon, it might be better described as dissociation, as both protein and polymer (to some extent) are removed from the complex.

Needleless to say that the opposite reaction – binding of the released SOD1 with the polycation corona must also proceeds in our system. Therefore, the effects of the ELF MF would perhaps be the greatest when synthetic or biological absorbents outside of the s-MNP complex capture the released protein molecules.

This consideration albeit simplified can also explain the trends for the effects of the field strength and frequency observed in the present study. As the field strength ***H*** increases the *F*_*HD*_ also increases. However, increasing ***H*** above certain value, that is sufficient to turn MNPs to 180° along with the changing field vector direction, cannot increase any further the rate of the SOD1 release since the system’s limiting states would be realized already. That should explain a saturation of the release (or a catalytic activity change) effect on the field strength. Increasing the frequency of the field first leads to the increase of the release rate, because the angular velocity of MNPs rotation in the field and *F*_*HD*_ value are also increasing. Moreover, the number of the cycles of the action of the force that prompts the SOD1 molecule migration towards the periphery of the corona also increases as the frequency of oscillations of MNP increases. However, above certain characteristic frequency the amplitude of the rotational-vibrational oscillation of MNPs decreases due to the “dragging” effect of the viscous media, resulting in the decrease of the nano-magneto-mechanical effects^17^. Previously, the peak frequency of 120 Hz was measured for the Brownian relaxation of protein coated 10 nm magnetite MNPs clusters with *D*_*HD*_ ≈ 60 nm exposed to ELF MF (amplitude of 10 Oe). Moreover, it was shown that the peak frequency shift is consistent with a decrease of the D_m_/D_HD_ ratio. The decrease of D_m_/D_HD_ from 1/5 to 1/6 lead to the frequency shift from 210 to 120 Hz^33^. Assuming similar dependence of the peak frequency on the D_m_/D_HD_, the critical frequency of our system which consists of 3–5 9 nm MNPs clusters (*D*_*HD*_ ≈ 100 nm, D_m_/D_HD_ ~1/7) should be in the range of 65–70 Hz. The measured characteristic frequency was indeed 50 Hz.

The antioxidant polyion complexes of SOD1 with PLL-PEG have shown recovery improvement in the models of ischemic brain injury and spinal cord injury^34,35^. However, due to the narrow time window for antioxidant enzymes to work in the acute phase of diseases and mitigate the oxidative stress, it is essential to use formulations with high catalytic activity and rapid enzyme release. Rotational oscillation motion of MNPs in the field generates a hydrodynamic force upon macromolecules in the complex, that promotes migration of the SOD1 to the complex periphery and SOD1 release. Therefore, combination treatments with magnetic s-MNPs/SOD1 complexes and ELF-MF application may first activate SOD and enhance efficacy and second, control the release of SOD1 at the disease site and, as a result, increase the catalytic and therapeutic efficacy of the formulation. The optimal exposure time is approximately 15 min, however the response is observed after as early as 1 min of exposure, suggesting that the approach is clinically applicable. This approach requires a use of specifically designed magnetic coils of various shapes and sizes, that can be tuned depending on the therapeutic target. For example, small coil with a diameter of several centimeters can be used for a localized exposure to AC magnetic field with the depth of penetration of ∼1 cm^3^ ^16^. For other applications or full body exposure, instruments similar to those designed and commercially developed for magnetic hyperthermia applications could be developed^36^. Thus, future studies should explore potential applications of the nano-magneto-mechanical actuation effect for the design of drug release systems and other functional healthcare materials.

## Materials and Methods

### Materials

Iron (III) acetylacetonate (Fe(acac)_3_), benzyl alcohol, SOD1, 3- (2-pyridyl)-5,6-diphenyl-1,2,4-triazine-4,4-disulfonic acid, sodium salt, pyrogallol (PG), 4-phenylspiro-[furan-2(3 H),1-phthalan]-3,3′-dione (Fluram), 4-(2-hydroxyethyl)-1-piperazineethanesulfonic acid (HEPES) and tris(hydroxymethyl)aminomethane (Tris) were purchased from Sigma Aldrich (USA). PLL-PEG with different PLL block lengths (10, 50, 100 r.u.) and constant PEG block (5 kDa) were from Alamanda Polymers (USA).

### Synthesis of PLL-PEG coated s-MNPs

Magnetite MNPs were synthesized according to the previously published procedure^25^. Briefly, 2.14 g of Fe(acac)_3_ and 45 ml of benzyl alcohol were placed in the three-neck flask. The reaction mixture was purged with argon, heated slowly to 100 °C, kept at this temperature under constant stirring for 1 h, gradually heated to 207 °C and, finally, incubated at this temperature for additional 40 h. After completion of this procedure the reaction mixture was slowly cooled down to the room temperature (RT) and mixed with anhydrous acetone. The MNPs were separated magnetically, washed with acetone and then dried on a rotary evaporator to completely remove the solvent. After that 20 mg MNPs were dispersed in 10 mL of alkaline water (pH 11–12), sonicated for 20–30 min, and then supplemented drop-wise with 10 mL alkaline solution of 4 mg/mL PLL-PEG under constant stirring. The mixture was stirred at RT for 17–20 h. To remove the excess of the unbound polymer, the solution was dialyzed against distilled water for 48 h, washed by centrifugal filtration using cellulose filters with 100 kDa cut-off and 10 mM HEPES buffer, pH 7.4 as eluent, and lyophilized.

### Polyion complexation of SOD1 to s-MNPs

In order to immobilize the enzyme on MNPs 2 mL of the 80 to 800 µg/mL SOD1 solution in 10 mM HEPES pH 7.4 were added dropwise under constant stirring to 2 mL of PLL-PEG coated s-MNPs dispersed in the same buffer with net concentration of 10^17^–10^18^ PLL amino groups/mL. The concentration of amino groups was measured with Fluram reagent (λ_ex_ = 390 nm, λ_em_ = 475 nm). The molar ratios of PLL amino groups to the carboxyl groups of SOD1 in the mixture was estimated assuming 38 carboxyl groups per one SOD1 molecule according to the Protein Data Base (PDB). The mixture was incubated for 15 min. The s-MNPs/SOD1 complexes were separated from the free enzyme by repeated centrifugal filtration from approx. 4 mL 10 mM HEPES media using cellulose filters with 100 kDa cut-off. After each filtration cycle the volume of the nanoparticle concentrate was approx. 1.5 mL. The procedure was repeated three times until no protein was detectable in the filtrate by the Bradford method^37^.

### Analytical procedures

The concentrations of Fe_3_O_4_ and SOD1 in the dispersions were determined by inductively coupled plasma mass spectrometry (ICP-MS) by measuring the content of and Fe^3+^, and Cu^2+^, respectively. Prior to the analysis each sample aliquots (100 µL) were added to a 5 mL glass vial, containing 100 µL of concentrated nitric acid and allowed to stand in a water bath at 60 °C overnight to release the copper cations and to dissolve magnetite. When indicated the Fe_3_O_4_ concentration was determined using a colorimetric assay. Briefly, 10 µL of MNPs sample solution was supplemented with 90 µL of concentrated hydrochloric acid and incubated at 60 °C for 2 h to dissolve the iron oxide. The sample solution was then cooled to RT, serially diluted with distilled water, and 325 µL of the resulting sample was mixed with 25 µL of 3-(2-pyridyl)-5,6-diphenyl-1,2,4-triazine-4,4 ‘ disulfonic acid sodium salt in deionized water (DI water) (Werner EasyPure 2). The Fe^3+^ concentration was determined spectrophotometrically (λ_abs_ = 490 nm) using standard solutions of iron for the calibration curve. When indicated the PLL-PEG and SOD1 relative content in the samples was analyzed by EI MS on a Bruker maXis Impact mass spectrometer with ion source Bruker Apollo II electrospray source at a flow rate of 180 µL/h. For this purpose, formic acid (Sigma # 94318) was added to all samples to a final concentration of 0.1%. Ion source settings were selected as follows: voltage – 4500 V, dry gas flow rate – 8 L/min, dry gas temperature 250 °C, Nebulizer pressure – 1 bar. Obtained MS1 spectra were analyzed in the Bruker Data Analysis software.

### Materials characterization

The phase analysis of the MNPs was carried out as described previously^38^ using a custom-made Mössbauer spectrometer (Faculty of Chemistry, M. V. Lomonosov Moscow State University), equipped with a nitrogen cryostat, and a moving source of 57Co(Rh) with activity 1.8 × 109 Bq at RT. The spectra were measured in transmission mode in the cryostat at 300 K and 78 K and recorded in 4096 channels. The spectrometer was calibrated against α-Fe. The transmission electron microscopy (TEM) was carried out as described previously^22^ with no staining using JEM 2010(HC), 200 kV (JEOL Ltd., Japan). TEM specimens were prepared by aspirating the samples diluted (1000 times) with DI water onto a copper TEM grid. The average diameter of the MNPs grains was calculated from TEM images by analyzing at least 300 MNPs for each sample using ImageJ software (National Institutes of Health, USA). The z-average effective hydrodynamic diameter of the particles (D_HD_), PDI and ζ-potential in aqueous dispersions were determined by DLS using ZetaSizer Nano ZS (Malvern). The samples were diluted 100 times and filtered through Millex-GP Syrnge filter unit, 0.22 µm (EDM Millipore) 1–2 min before measurements. TGA of the polymer-stabilized s-MNPs was determined using Q50 (TA Instruments, DE) as described previously^25^. 5−10 mg of the lyophilized unloaded-formulation was dried in the furnace at 110 °C in order to remove the moisture followed by steady heating at 5 °C/min to 1000 °C. The thermogram was analyzed using the Universal Analysis software (TA Instruments, DE) to deduce the total loss of the organic components and amount of iron oxide per mg of MNPs. The saturation magnetization (M; emu/g) of the bare MNPs and s-MNPs was determined as previously described^25^ by superconducting quantum interference device (SQUID) analysis (Quantum Design) Physical Measurement System (PPMS) equipped with vibration magnetometric device (VSM) with 2 nm amplitude of oscillation, frequency of 40 Hz at 300 K.

### The effect of an alternating magnetic field on s-MNPs/SOD1 complexes

Exposure of samples with ELF MF was conducted, using instruments, constructed by “Nanodiagnostics” LLC (Russia). ELF MF was homogeneous with an accuracy of ±2%, i.e. it had the gradient less than 20 mT/m and, as a result, it causes only rotational oscillations of MNPs and negligible translational ones. The unit comprises LF current generator with adjustable power (up to1.5 kW) and water-cooled inductor. MF frequency could be varied in the range of 30 to 3000 Hz. The unit is equipped with the thermostated working cell and controller that allows setting the necessary temperature and exposure times in the ELF MF and the pause between them. Experiments were conducted in the frequency range of 30–160 Hz and the magnetic field strength was varied from 8 to 120 kA/m. 50 µL of sample with SOD1 were placed in a 1 mL cuvette, and 900 µL of Tris buffer (50 mM, pH = 8.2) were added to get the SOD1 final concentration of 25–60 ng/mL, that corresponds to enzyme activity of 20–60% of inhibition of PG autoxidation. The cuvette was placed in the sample compartment of the device for AMF exposure, and incubated for a certain time period (30 sec., 5, 10, 20, 15, 25 min). The temperature of the working sample compartment was kept at 25 °C and under any experimental conditions did not vary more than a few tenths of a degree during the incubation in the ELF MF.

After the field was turned off, 50 µL of 0.5 mg/mL PG solution were added to the cuvette, and the autoxidation reaction kinetics was monitored using SpectraMax M5 instrument at λ_abs_ = 420 nm. In other words, here we studied the effect of ELF MF after exposure on the SOD1 activity and those residual changes in the macromolecule structure (due to deformation and/or deimmobilization).

Percent inhibition of autoxidation was determined using the following formula:

1 $$inhibition, \% =\frac{(Sp-Ss)}{Sp}\,\ast \,100$$

where S_p_ – the slope of the kinetic reaction of PG autoxidation curve in the absence of SOD1; S_s_–the slope of the kinetic reaction curve PG autoxidation in the presence of SOD1. The operating range of SOD1 concentrations lies in the interval corresponding to 20–80% of the PG autoxidation inhibition.

To examine desorption of the macromolecules from s-MNPs and s-MNPs/SOD1 complexes under exposures to ELF MF 500 µL of sample in 10 mM HEPES buffer, pH = 7.4 were placed in the centrifugal filters with 100 kDa cut-off pore size (Mw SOD1 32 kDa, Mw polymer 16 kDa). One filter was placed in the sample compartment of the device for ELF MF exposure (55 kA/m 50 Hz) and incubated for 5 or 25 min. Then both samples were quickly placed in a centrifuge and spun for 5 minutes at 4500 g at RT. Next, the ratio of Cu^2+^/Fe^3+^concentrations in the concentrated solutions were measured by ICP-MS, amount of PLL_100_-PEG and SOD1 in the filtrates were measured by EI MS and the catalytic activity of SOD1 in filtrates was determined as percent inhibition of the reaction of PG autoxidation.

### Statistical analysis

Statistical analyses were performed using Origin 9.0 (OriginLab Corporation, Northampton, Massachusetts, USA). All data were analyzed using a one-way ANOVA. Where applicable, reported p-values have been adjusted for multiple comparisons using the Ryan-Einot-Gabriel-Welsch post-hoc method. Significance was reported for p < 0.05.

## Supplementary information

Supplementary information.
