# Supplementary material for: Enzyme Release from Polyion Complex by Extremely Low Frequency Magnetic Field
Source: Sci Rep. 2020 Mar 16;10:4745. doi: 10.1038/s41598-020-61364-w (PMC7076007; doi:10.1038/s41598-020-61364-w)
Supplement: Supplementary file 1 — Supplementary information. [file 41598_2020_61364_MOESM1_ESM.pdf]

Supporting Information

ENZYME RELEASE FROM POLYION COMPLEX BY EXTREMELY LOW FREQUENCY MAGNETIC FIELD

Kseniya Yu.Vlasova[1], *Hemant Vishwasrao*[2], *Maxim A.Abakumov*[3,4], *Dmitry Yu.Golovin*[5], *Sergey L.Gribanovsky*[5], *Alexander O.Zhigachev*[5], *Andrey A.Poloznikov*[1], *Alexander G.Majouga*[1,3,6], *Yuri I.Golovin*[1,5], *Marina Sokolsky-Papkov*[2], *Natalia L.Klyachko*[1,2,5], *Alexander V. Kabanov**[1,2]

[1] *Laboratory for Chemical Design of Bionanomaterials, School of Chemistry, Lomonosov Moscow State University, Moscow, 119991, Russia*
[2] *Center for Nanotechnology in Drug Delivery and Division of Molecular Pharmaceutics, Eshelman School of Pharmacy, University of North Carolina at Chapel Hill, NC 27599, U.S.A.*
[3] *National University of Science and Technology MISIS, Moscow, 119049, Russia*
[4] *Department of Medical Nanobiotechnology, Pirogov Russian National Research Medical University, Moscow, 117997, Russia*
[5] *G.R. Derzhavin Tambov State University, Tambov, 392036, Russia*
[6] *D. Mendeleev University of Chemical Technology of Russia, Moscow, 125047, Russia*

E-mail: kabanov@email.unc.edu

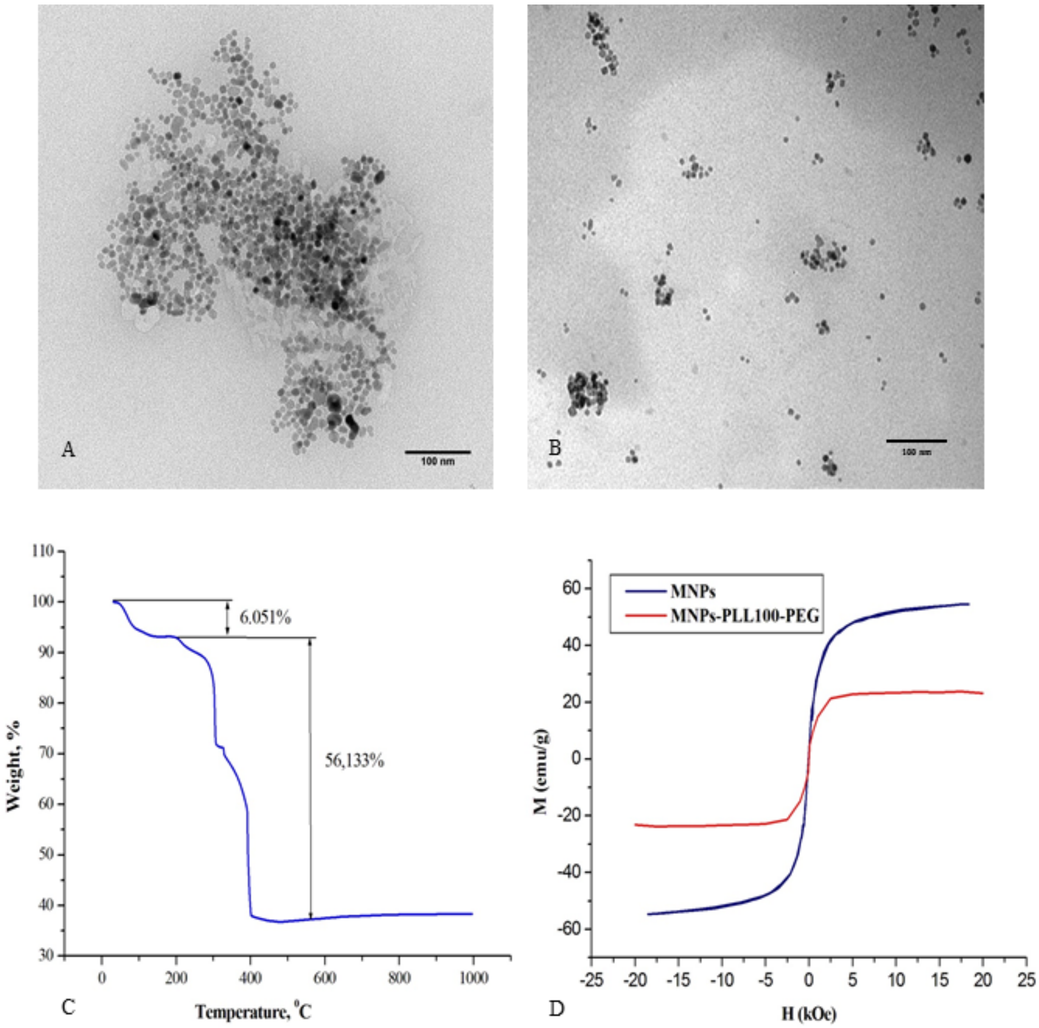

Figure S1. TEM microphotograph of **(A)** bare MNPs, and **(B)** PLL_{100}-PEG block copolymer coated MNPs and **(C)** TGA and **(D)** saturation magnetization of PLL_{100}-PEG block copolymer coated MNPs. MNPs are produced via thermal decomposition of $Fe(acac)_3$ in benzyl alcohol. Samples were dispersed in **(A)** alkaline water (pH 11-12) and then diluted 100 times in distilled water or **(B-C)** distilled water.

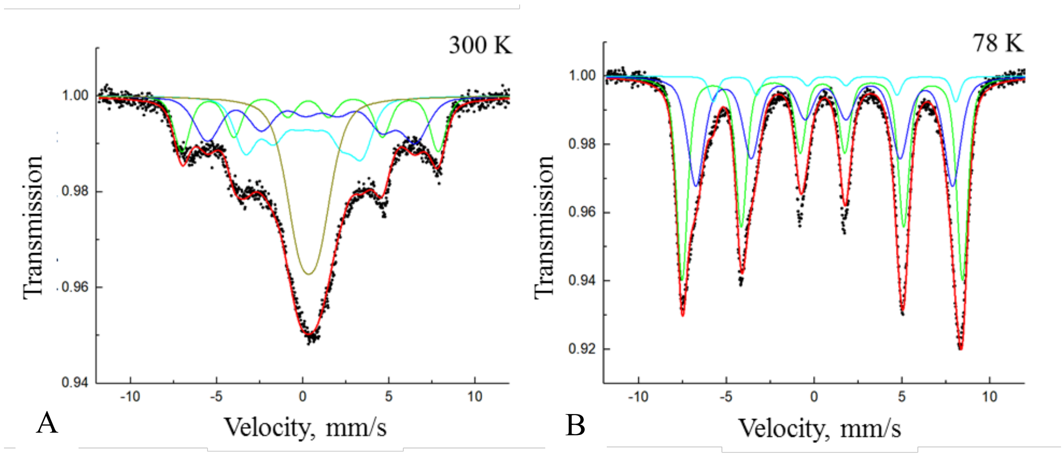

Figure S2. Mössbauer spectra (black dots) recorded at **(A)** 300 K and **(B)** 78 K for MNPs sample. The red solid line is the best fit to the experimental data, and the colored lines show each component of the total fit.

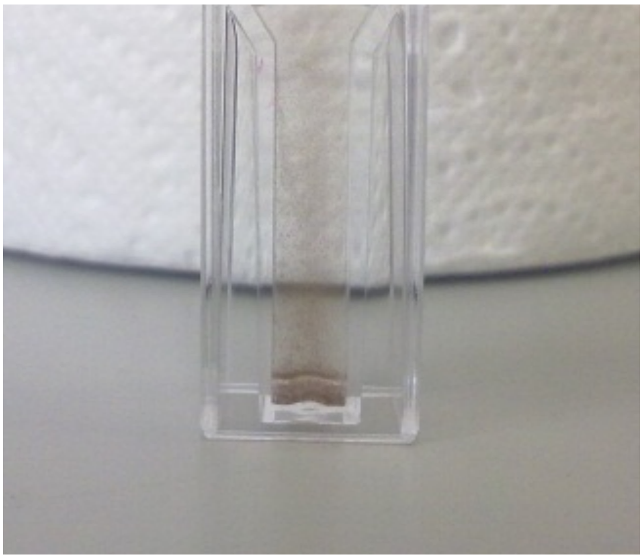

Figure S3. Aggregation and precipitation of the MNPs/PLL_{10}-PEG/SOD1 complex during preparation (in 30 min) in 10 mM HEPES, pH 7.4 at RT.

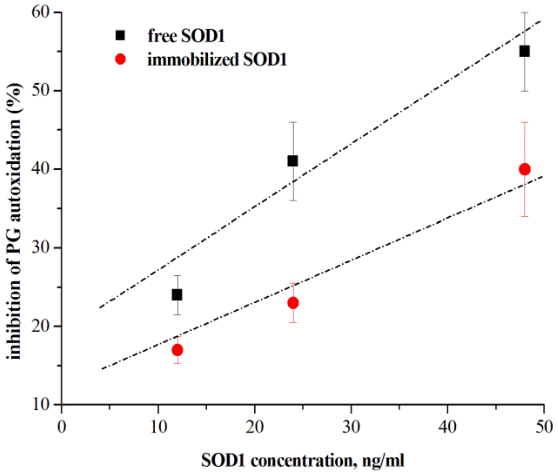

Figure S4. Catalytic activity of SOD1 before and after its immobilization on s-MNP at various enzyme concentrations. The concentrations of Fe_3O_4 in each point are constant (50 ng/mL). The enzymatic activity was measured by the inhibition of the reaction of autoxidation of PG in the presence of SOD1. After enzyme immobilization on the s-MNPs the free SOD1 was not removed. Data are presented as mean ± SD (n=3). The data show that percent inhibition of PG autoxidation for the immobilized enzyme is less in comparison to that for the enzyme free form at the same concentration, i.e., enzyme activity is decreased.

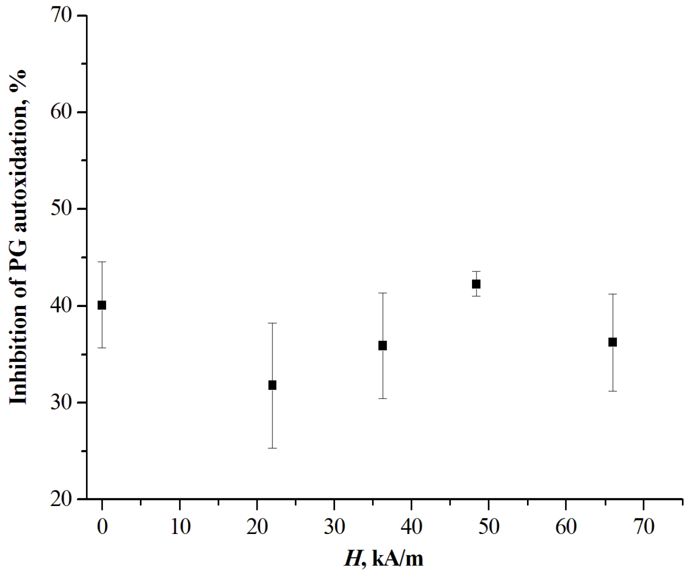

Figure S5. Catalytic activity of free SOD1 after its exposure to ELF MF of 50 Hz frequency for 30 min. [SOD1] = 24 ng/mL. 50 mM Tris-HCl buffer, pH 8.2 at RT. Data are presented as mean ± SD (n = 3).

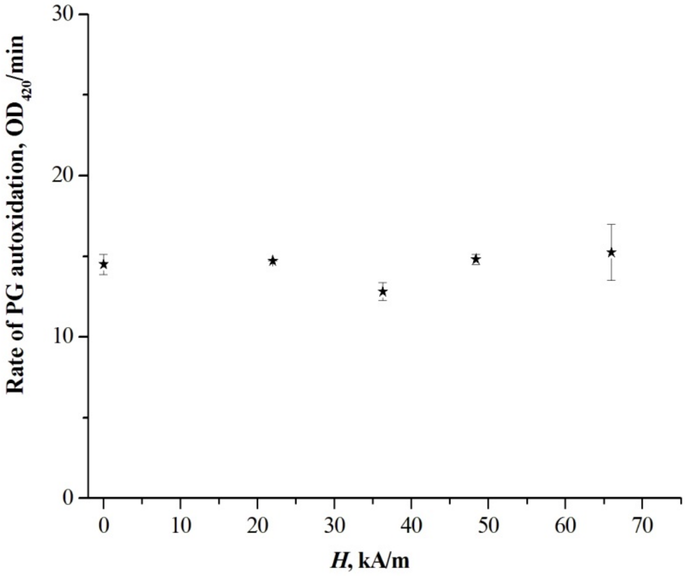

Figure S6. The rate of PG autoxidation in the presence of MNPs/PLL_{100}-PEG after the solution exposure to ELF MF of 50 Hz frequency for 30 min. [Fe_3O_4] = 50 ng/mL. 50 mM Tris-HCl buffer, pH 8.2 at RT. Data are presented as mean ± SD (n = 3).

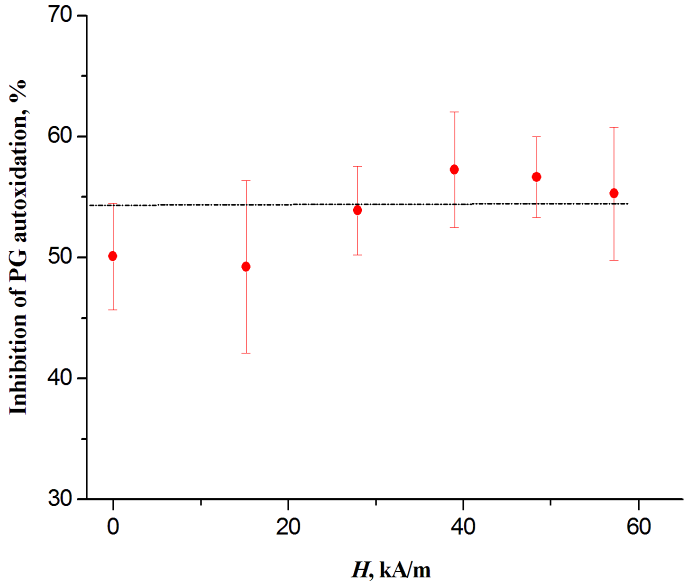

Figure S7. Effect of the ELF MF on SOD1 enzymatic activity of the MNPs/PLL_{50}-PEG/SOD1 complex at the constant field frequency of 50 Hz and varying field intensity. The samples were exposure to the ELF MF for 30 sec at RT. The Fe_3O_4 and SOD1 concentrations were 50 ng/mL and 50 ng/mL, respectively. The dashed line represents the SOD1 activity MNPs/PLL_{50}-PEG/SOD1 complex without ELF MF exposure. The samples were dispersed in 50 mM Tris-HCl buffer, pH 8.2. Data are presented as mean ± SD (n = 3); differences between treatments are not significant.

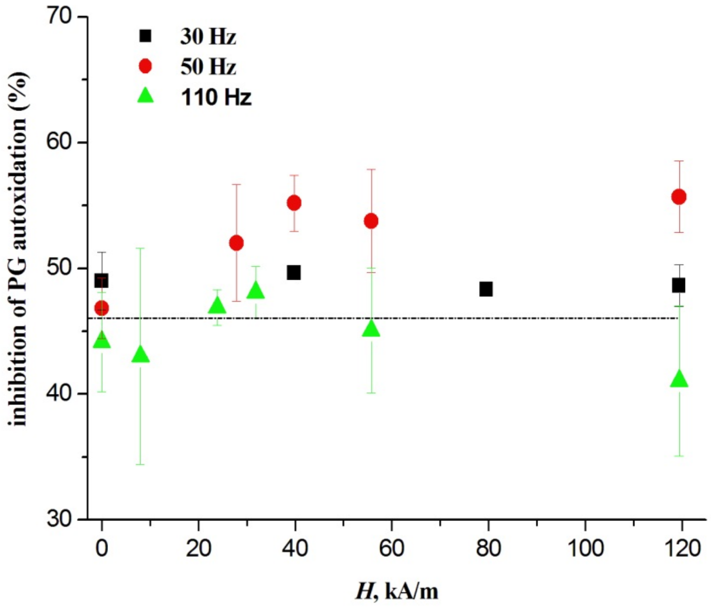

Figure S8. Effect of ELF MF on the SOD1 enzymatic activity of the MNPs/PLL_{100}-PEG/SOD1 complex at the field frequencies of 30, 50 and 110 Hz and varying field intensity. The samples were exposed to the ELF MF for 30 sec at RT. The Fe_3O_4 and SOD1 concentrations were 54.6 ng/mL and 50 ng/mL. The samples were dispersed in 50 mM Tris-HCl buffer, pH 8.2. Data are presented as mean ± SD (n = 3).

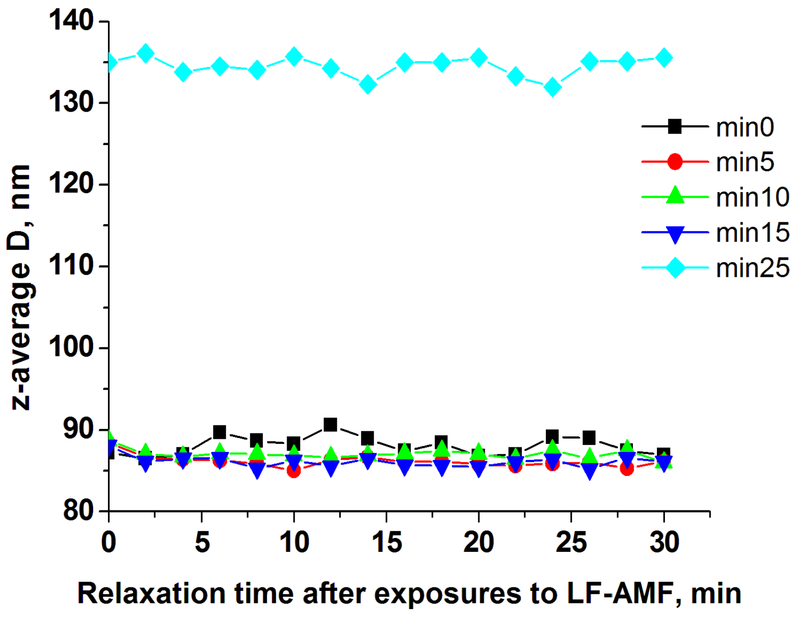

Figure S9. Hydrodynamic diameter MNPs/SOD1 complex at different times (0, 5, 10, 15, and 25 min) after exposure to ELF MF (50 Hz, 55 kA/m). [Fe_3O_4] = 11.5 µg/mL. 10 mM HEPES buffer (pH 7.4).

Table S1. Increase in the SOD1 activity in the filtrate after exposure of the MNP-PLL_{100}-PEG-SOD1 complex dispersion to ELF MF (f = 50 Hz, H = 55 kA/m).[a)]

Exposure duration, min	Inhibition of PG autoxidation (%)	
	no ELF MF	after ELF MF
0.5	17±5	19±5
1	17±5	23±4
5	17±5	27±3

[a)] During the exposure of the MNP-PLL_{100}-PEG-SOD1 dispersion to the ELF MF the Fe_3O_4 and SOD1 concentrations were 43.6 µg/mL and 49.2 µg/mL, respectively. Following the exposure, the MNP-PLL_{100}-PEG-SOD1 samples were concentrated via centrifugal filtration (cut-off 100 kDa) and SOD1 activity in the filtrates was determined as percent inhibition of the reaction of PG autoxidation. Data are presented as mean ± SD (n=3).

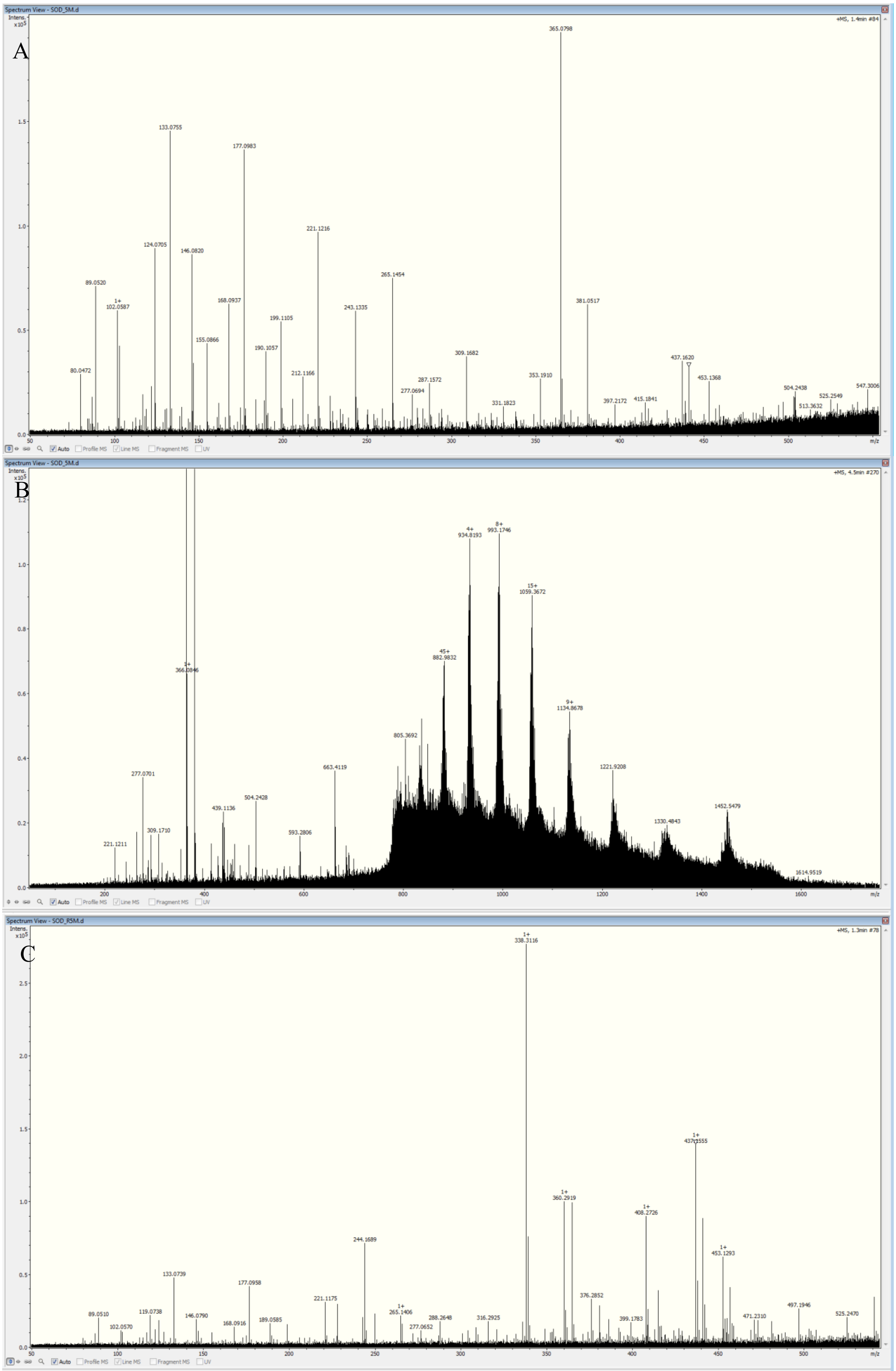
Spectrum View - SOD_5M.d
A
+MS, 1.4min #84
365.0798
133.0755
177.0983
221.1216
124.0705
146.0820
89.0520
1+
102.0587
168.0937
265.1454
381.0517
243.1335
199.1105
155.0866
190.1057
309.1682
437.1620
80.0472
212.1166
353.1910
453.1368
287.1572
277.0694
331.1823
397.2172
415.1841
504.2438
547.3006
525.2549
513.3632
Spectrum View - SOD_5M.d
B
+MS, 4.5min #270
4+
934.8193
8+
993.1746
15+
1059.3672
45+
882.9832
1+
366.0846
9+
1134.8678
805.3692
663.4119
277.0701
1221.9208
504.2428
439.1136
1452.5479
309.1710
1330.4843
593.2806
221.1211
1614.9519
Spectrum View - SOD_R5M.d
C
+MS, 1.3min #78
1+
338.3116
1+
437.1555
1+
360.2919
1+
408.2726
244.1689
1+
453.1293
133.0739
177.0958
221.1175
376.2852
497.1946
1+
265.1406
89.0510
119.0738
146.0790
288.2648
316.2925
399.1783
471.2310
525.2470
102.0570
168.0916
189.0585
277.0652
Intens.
m/z

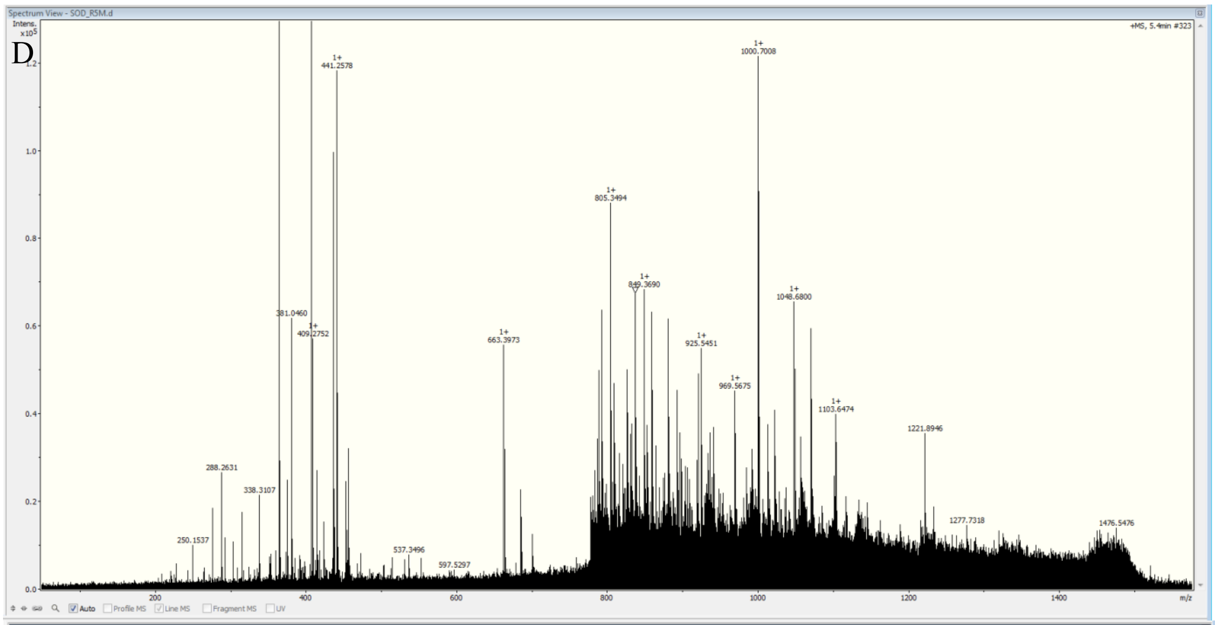
Spectrum View - SOD_RSM.d
D
+MS, 5.4min #323
Intens. x10^5
1000.7008
441.2578
805.3494
849.3690
1048.6800
381.0460
409.2752
663.3973
925.5451
969.5675
1103.6474
1221.8946
288.2631
338.3107
250.1537
537.3496
597.5297
1277.7318
1476.5476
m/z

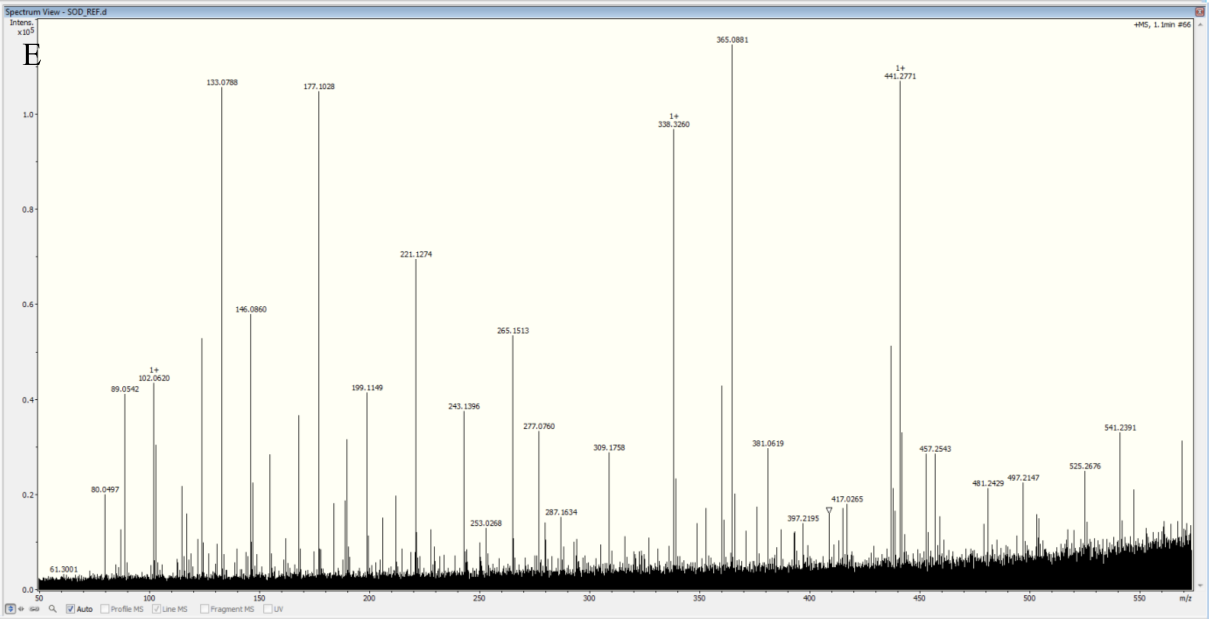
Spectrum View - SOD_REF.d
E
+MS, 1.1min #66
Intens. x10^5
365.0881
441.2771
133.0788
177.1028
338.3260
221.1274
146.0860
265.1513
102.0620
89.0542
199.1149
243.1396
277.0760
309.1758
381.0619
457.2543
541.2391
525.2676
497.2147
481.2429
80.0497
417.0265
253.0268
287.1634
397.2195
61.3001
m/z

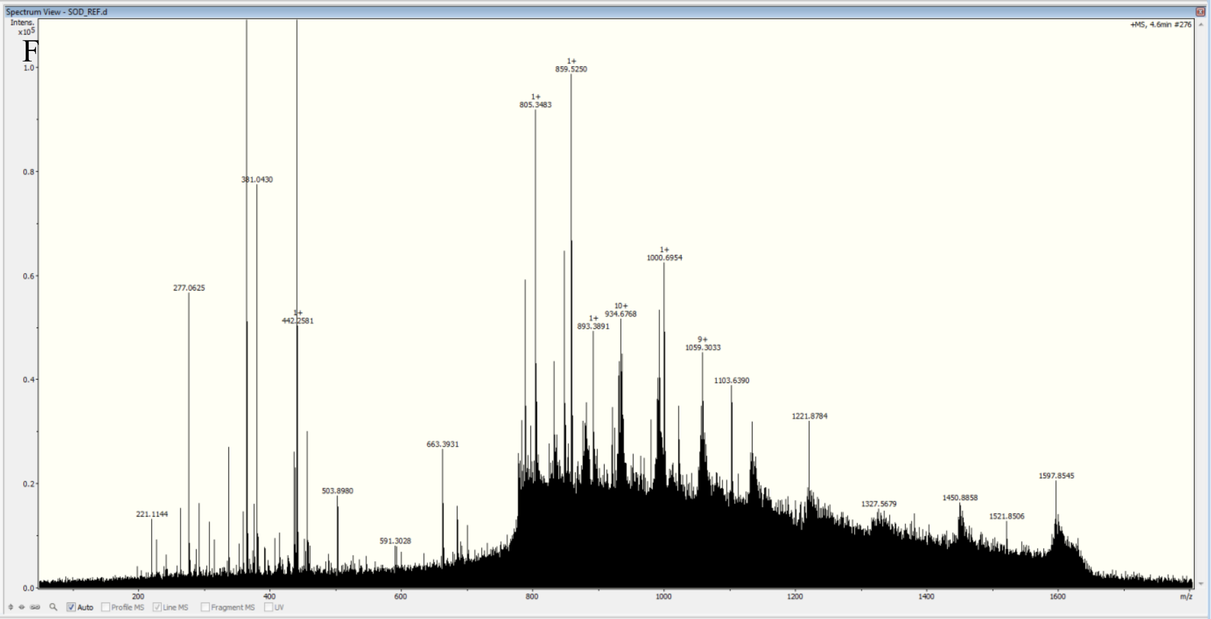
Spectrum View - SOD_REF.d
F
+MS, 4.6min #276
Intens. x10^5
859.5250
805.3483
381.0430
1000.6954
277.0625
442.2581
934.6768
893.3891
1059.3033
1103.6390
1221.8784
663.3931
1597.8545
503.8980
221.1144
591.3028
1327.5679
1450.8858
1521.8506
m/z

Figure S10. EI MS analysis. Ion source settings were selected as follows: voltage – 4500V, dry gas flow rate - 8 L/min, dry gas temperature 250°C, Nebulizer pressure - 1 bar. Obtained MS1 spectra were analyzed in the Bruker Data Analysis software. Samples: filtrates of centrifuged MNPs-PLL_{100}-PEG-SOD1 nanocomplex without (control) and after 5 min or 25 min exposures of ELF MF (50 Hz, 55 kA/m). **A** - Spectrum of PEG for sample after 5 min exposure of ELF MF; **B** - Spectrum of SOD1 for sample after 5 min exposure of ELF MF; **C**- Spectrum of PEG for sample after 25 min exposure of ELF MF; **D** - Spectrum of SOD1 for sample after 25 min exposure of ELF MF; **E** - Spectrum of PEG for control sample; **F** - Spectrum of SOD1 for control sample.

Golovin et al[1] reported that for a spherical particle of D_m = 10 nm in aqueous solution, the characteristic thermal time $t^* = D_m^2/4\chi$ of heat diffusion in nonstationary heating mode is 11-12 orders of magnitude smaller than a typical exposure time in our experiments (0.5-25 min) ($\chi = \lambda/c\rho$ – thermal diffusivity, c – heat capacity, λ – thermal conductivity coefficient of the surrounding medium accordingly). Consequently, already after ~ 1 ns after application of ELF MF the temperature field in the vicinity of MNPs ceases to be adiabatic, and a few tens of nanoseconds later becomes quasi-stationary. (It is obvious that the exact values of χ and D_m have no impact on this fundamentally important conclusion.) In the quasistationary mode, the value of local overheating ΔT on MNPs surface relatively to the surround medium is determined by the equation:

$$\Delta T_s = qD_m^2\rho_m/(12\lambda) \quad (1),$$

where ρ_m - density of the magnetic core. A typical value for q is 100 - 1000 W/g in a regular field with H = 20 - 30 kA/m and f = 200 - 500 kHz.[2] Since $q \sim fH^2$, for our conditions (f = 30 - 400 Hz and $H \approx$ 100 kA/m) value of q is 1 - 10 W/g. At such low values of q, as well as R_m = 3-10 nm and $\lambda \approx$ 0.6 W/(m·K) the ΔT value, according to (1), is not more than 10^{-9} - 10^{-8} K. For the aggregates of 10-100 particles ΔT in the center of the complex will be about 10^{-8} - 10^{-7} K. Overall, at any reasonable values of the variables q, D_m, λ, and formation of aggregates of hundreds of MNPs, the amount of heat even in the center of large complex of MNPs is negligible in our experiments. Therefore, the initial assessment of the influence of AMF on

our system was evaluated by measuring changes of enzyme activity, which, if heating is excluded, can only occur as a result of some mechanical processes.

References

[1] a) Y.I. Golovin, N.L. Klyachko, A.G. Majouga, D.Y. Golovin1, S. Gribanovsky. IOP Conf. Series: Materials Science and Engineering, **2015**, 98, 012016. b) Y.I. Golovin, S.L. Gribanovsky, D.Y. Golovin, A.O. Zhigachev, N.L. Klyachko, A.G. Majouga, M. Sokolsky, A.V. Kabanov, *J Nanopart Res*. **2017**, 19,1.
